# Adaptive feature interaction enhancement network for text classification

**DOI:** 10.1038/s41598-025-95492-y

**Published:** 2025-04-03

**Authors:** Rui Su, Shangbing Gao, Kefan Zhao, Junqiang Zhang

**Affiliations:** 1 School of Computer and Software Engineering, Huaiyin Institute of Technology, Huai’an, Jiangsu China; 2 Jiangsu Internet of Things Mobile Internet Technology Engineering Laboratory, Huai’an, Jiangsu China

**Keywords:** Natural language processing, Text classification, Adaptive feature enhancement, Interaction gate, Pre-training, Computational science, Computer science, Scientific data, Information technology

## Abstract

Text classification aims to establish text distinctions, which face difficulty in capturing global text semantics and local details. To address this issue, we propose an Adaptive Feature Interactive Enhancement Network (AFIENet). Specifically, AFIENet uses two branches to model the text globally and locally. The adaptive segmentation module in the local network can dynamically split the text and capture key phrases, while the global network grasps the overall central semantics. After obtaining the results from the two branches, an interaction gate is designed to evaluate the confidence of the global features and selectively fuse them with the local features effectively. Finally, the interactively enhanced features are re-input into the classifier to improve text classification performance. Experiment results show that our proposed method can effectively enhance the performance of backbone networks such as TextCNN, RNN, and Transformer with fewer parameters. AFIENet achieved an average accuracy of 3.82% and an F1-score of 3.88% improvement across the three datasets when using Transformer as the backbone network. The comparable results to MacBERT that obtained with static word vectors also reflect the applicability of the proposed method.

## Introduction

Text classification^1^ is a key natural language processing task widely used in areas such as news classification^2^, sentiment analysis^3^, topic labeling^4^, etc. Traditional machine learning methods rely on shallow feature extraction, lacking sufficient understanding of the underlying semantics, structure, sequence, and context within the text. Their limited representational abilities result in relatively low classification performance^5^. The advent of deep learning solves issues like the inferior ability of shallow architectures to represent complex functions, gradually replacing traditional machine learning as the mainstream approach in text classification^6^. However, deep learning models demonstrate inconsistent results when handling varying text lengths. However, there are also difficulties controlling model parameter size.

With the wide application of deep learning in natural language processing, numerous text classification models incorporating deep learning have been continuously proposed. Early convolutional neural networks (CNNs), originally applied in computer vision for their visual cortex-like structure, were soon widely adopted in text classification. Kim^7^ first applied CNNs through using convolutional kernels of varying scales to capture local sentence information and improve model interpretability. However, this also led to vanishing gradients. To address this issue, Wang et al.^8^ proposed the Deep Pyramid Convolutional Neural Network (DPCNN) with a pyramidal structure and layer hopping to mitigate gradient decay. Joulin et al.^9^ put forth the FastText model, projecting input text into an embedding space for quick, shallow convolutional learning and improved classification with lower computational costs. Convolutional networks are limited in modeling longer sequences due to small convolutional windows. Recurrent Neural Networks (RNNs) avoids this issue by treating sentences wholly as complete sequences. Thus, sequential models like RNN^10^ and Long Short Term Memory (LSTM)^11^ better capture overall text semantics. The Gated Recurrent Unit (GRU)^12^ was introduced to solve the exploding/vanishing gradient problems of RNNs to some extent through learned gating of information flow and propagation.

Most existing classification methods using CNNs and RNNs derive text representations through max-pooling of hidden layers or final states, potentially overlooking decisive keywords. Attention mechanisms can effectively learn global semantic information in text. Transformer^13^ employed multi-head self-attention to capture richer features, with attention weights measuring each part’s influence on the whole. Additionally, Bi-directional Encoder Representations from Transformer (BERT)^14^, a pre-trained bi-directional 12-layer Transformer model, better captures deeper contextual information. However, BERT’s^14^ stacked architecture and numerous attention mechanisms lead to exponential growth in parameters. Current models still need a better balance between modeling text length variability and parameter efficiency.

To address the limitations of previous methods, we propose an Adaptive Feature Interactive Enhancement Network (AFIENet) for text classification. AFIENet contains two network branches, i.e., a Global Feature Extraction Network (GE-Net), a Local Adaptive Feature Extraction Network (LA-Net) and along with an Interactive Enhancement Gate (IE-Gate). Specifically, GE-Net employs a feature extraction model to learn global feature from the input text to obtain a holistic representation. Meanwhile, LA-Net first performs adaptive segmentation of the text before extracting local feature. Then, the IE-Gate is proposed to select local features based on confidence levels to interactively fuse with the global feature, obtaining an enhanced semantic expression. Overall, the cooperation of global and local networks enables more comprehensive and focused feature representations, thus improving text classification performance.

Our contributions are as follows:

- We propose an Adaptive Feature Interactive Enhancement Network for text classification, which forms a dual-branch architecture and achieves complementary semantic representations to improve classification performance.
- In the local network branch, an adaptive text segmentation module is designed to perform the local feature extraction of text with different lengths.
- The interactive gate proposes to use a gating mechanism for feature selection fusion, which can effectively filter the noise of local features and specifically uses the confidence of global features to evaluate the enhancement, to avoid the influence of arbitrarily mixed local noise.

## Relared work

### Variable-length text processing

Many excellent modeling approaches have been proposed for the existing text classification tasks for variable-length texts and that with different semantic complexity. To address the complexity and diversity of text, Zhang et al.^15^ proposed to incorporate multi-granularity dynamic semantic representations for product text data in the chemical industry, where a Chinese pre-trained language model is used upstream of the classification task to obtain the dynamic word-vector language of the sentences, and where the idea of adversarial training is introduced to increase the robustness of the text representations. Aiming at the problems of short text feature sparsity and model dimension explosion, Yang et al.^16^ proposed a short text classification model using the attention mechanism and the word symbiosis of labeled graphs, which solved the problems of short text sparsity and topic text heterogeneity. Li et al.^17^ fused knowledge perception and dual attention mechanism, which enabled the classification model to improve the efficiency of model information extraction while acquiring semantic features of short text.

On the task of performing long text classification, direct long sequence processing will ignore the hierarchical information contained in the document, to solve this problem, SUN^18^ performs multi-layer modeling according to the hierarchy of the text, as well as using the hybrid attention mechanism to obtain the obvious category features in the hierarchy, which enables the model to better capture the important information of the document and further improves the accuracy of the long document classification. Zhang et al.^19^ proposed a fusion multi-feature text classification method in the face of long text classification and constructed a classification model that fuses contextual, local, and averaged features, which effectively improves the accuracy in the task of long text classification. When targeting text datasets of varying lengths, Zheng et al.^20^ proposed a text classification method based on LDA, combining the LDA model to enrich the word vector information of the text, and adding a fusion layer inside the original convolutional neural network, which increases the richness of the feature representation, and solves the problem of insufficient feature representation resulting in a low accuracy rate of text classification.

Since Vaswani et al.^13^ proposed the Transformer model, it has significantly improved the performance of various natural language processing tasks, including machine translation, text generation, text summarization, etc. Many Transformer-based works^14,21,22^ have also made significant progress in variable length text processing tasks, such as Microsoft Research^23^ introduced relative position embedding and block masking to improve the model’s ability to handle long sequences. In addition, the explosive emergence of large models^24,25^ marks a new era in text processing.

While these methods have advanced text classification, most exhibit instability when handling varying text lengths and semantic complexity. A key reason is differing models focus on global versus local features, failing to balance semantic representation across short and long texts. This results in insufficient generalization on variable lengths. To enable adaptive processing, we propose an adaptive text segmentation layer for digital text modeling. This module divides the original text into segments based on adaptive lengths, dynamically adjusting segmentation granularity. Feature extraction is then performed separately on each segment. This mechanism can more effectively focus on key local features potentially overlooked when modeling entire sentences holistically.

### Gating units

In recurrent neural networks such as the LSTM, a large number of gating structures are used to control the flow and transfer of information, including the input gate, forgetting gate, and output gate. This mechanism can effectively avoid the gradient vanishing and gradient explosion problems and enhances the ability to model the long-term dependence of sequence data.

Similar gating designs have also been widely used in other network structures, for example, Ma et al.^26^ implemented feature screening through gating units and used gates to control the strength of information flow to the lower layers, thus mitigating gradient dispersion and improving the accuracy of model classification. Li et al.^27^ used gating components to map statistical features into a shared information space to make the two latent representations compatible with each other, thus assisting text classification to optimize the classification effect. Xu et al.^28^ proposed a new method for sentiment analysis and classification based on bidirectional gated recursive unit model. Yang et al.^29^ distinguished sequences and improved performance by integrating gated residual attention units and channel embedding techniques. In addition, gating units are often used in other network architectures to improve performance, such as recursive unit driven multi-dimensional dynamic graph neural networks.^30^.

### Attention mechanisms and pre-trained models

In recent years, attention mechanisms and pre-trained language models have demonstrated powerful modeling capabilities in natural language processing tasks. Attention mechanisms can effectively focus on the semantic core of the text, while pre-trained models have semantically rich word vector representations. Combining the two organically can further improve the effect of text comprehension and analysis. Yang et al.^31^ proposed the HAN (Hierarchy Attention Network) model with attention as a component of text classification, giving different attention to sentence-level and word-level scores, and selecting sentences and words containing helpful classification to improve classification performance. Subsequently, Cui et al.^32^ proposed a pre-trained language model MacBERT (MLM as correction BERT) for Chinese text, which greatly improved text classification performance. Zhan et al.^33^ utilized cross attention mechanism to focus text features with aspect category features, the model focuses on the features most relevant to the given aspect. In addition, there are also research methods in other fields such as medical analysis^34,35^ that combine pre-trained models for attention mechanisms. For example, Xiao et al.^36^ proposed an adaptive attention model that integrates domain knowledge and optimizes its ability to understand medical terminology and handle complex contexts.

Inspired by the above methods, our proposed framework also absorbs the ideas of attention and pre-training. We use dynamic word vectors after pre-trained to further enhance the semantic nature of text representation. Experimental results show that modeling with dynamic word vectors generated from the pre-trained model greatly improves the model’s effectiveness on the text classification task.

## Methodology

**Fig. 1:**
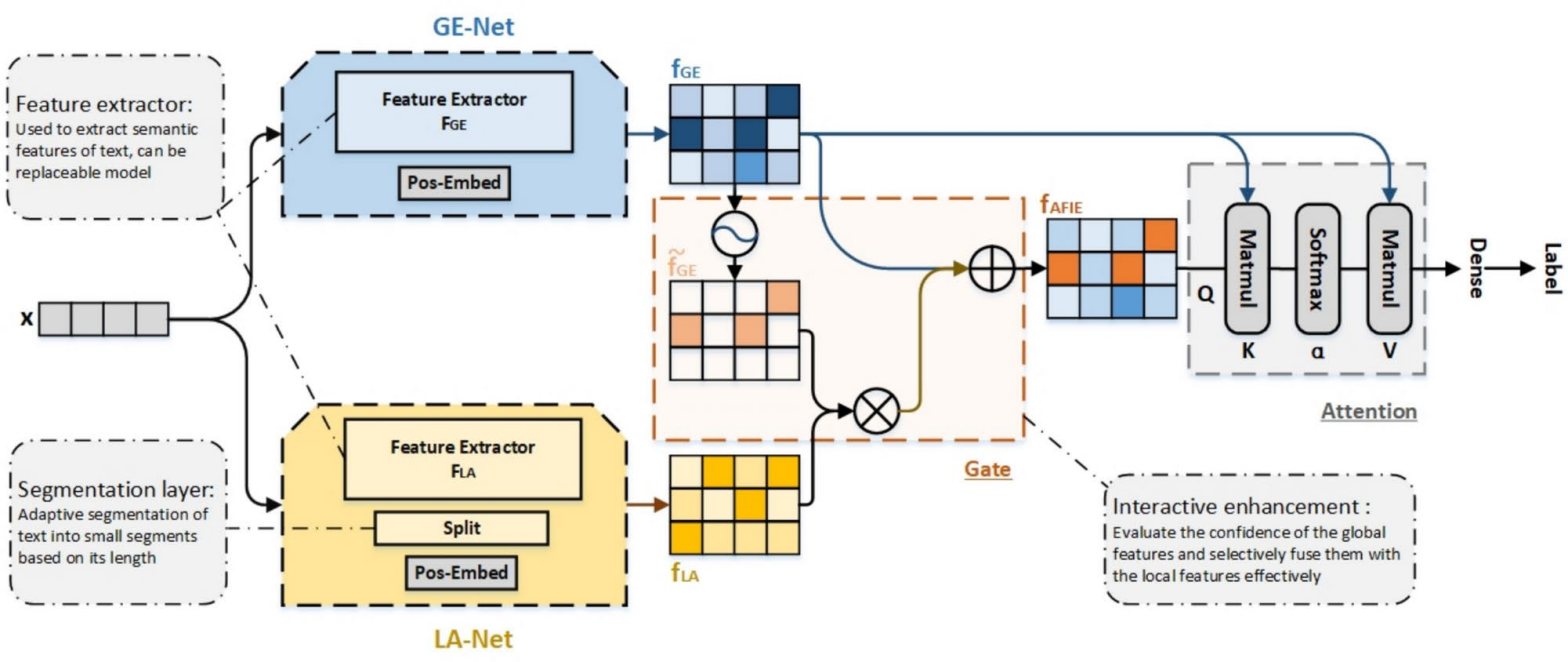
Structure of the adaptive feature interaction enhancement network (AFIENet). We used different colors to distinguish the features obtained by the two networks, i.e., GE-Net and LA-Net, to clearly illustrate the data flow of AFIENet.

In this article, we propose an Adaptive Feature Interactive Enhancement Network (AFIENet), whose structure is shown in Fig. 1. AFIENet contains three main components:

- Global feature extraction network, *i.e.*, GE-Net, which is used to learn global semantic information from the whole text;
- Adaptive local feature extraction network, *i.e.*, LA-Net, which can dynamically adjust the extraction range of local features according to the text length;
- Interaction Enhancement Module, *i.e.*, IE-Gate, which enhances the interaction between global and local features by evaluating the confidence level to filter out the noise.

Specifically, GE-Net and LA-Net model the text from global and local levels respectively, and use the feature extractors $F_{GE}$ and $F_{LA}$ to obtain the feature representations $f_{GE}$ and $f_{LA}$. GE-Net provides a high degree of flexibility by learning global semantic information from the entire input text, while LA-Net adapts to text length to capture local features dynamically. To obtain more adequate semantic information, IE-Gate first counts the confidence of the global features and then fuses them with the local features for weighted fusion. Below, we will provide a detailed description of each component of AFIENet.

**Feature Extractor**. Given a dataset $D=\{(x_i,l_i)\}_{i=1}^N$ , where $x_i$ is the text with input length *Seq* and $x_i$ is the label corresponding to the text $x_i$. It should be noted that GE-Net and LA-Net have the same input $x_i$ and feature extractors, but they do not share network parameters.

**Fig. 2:**
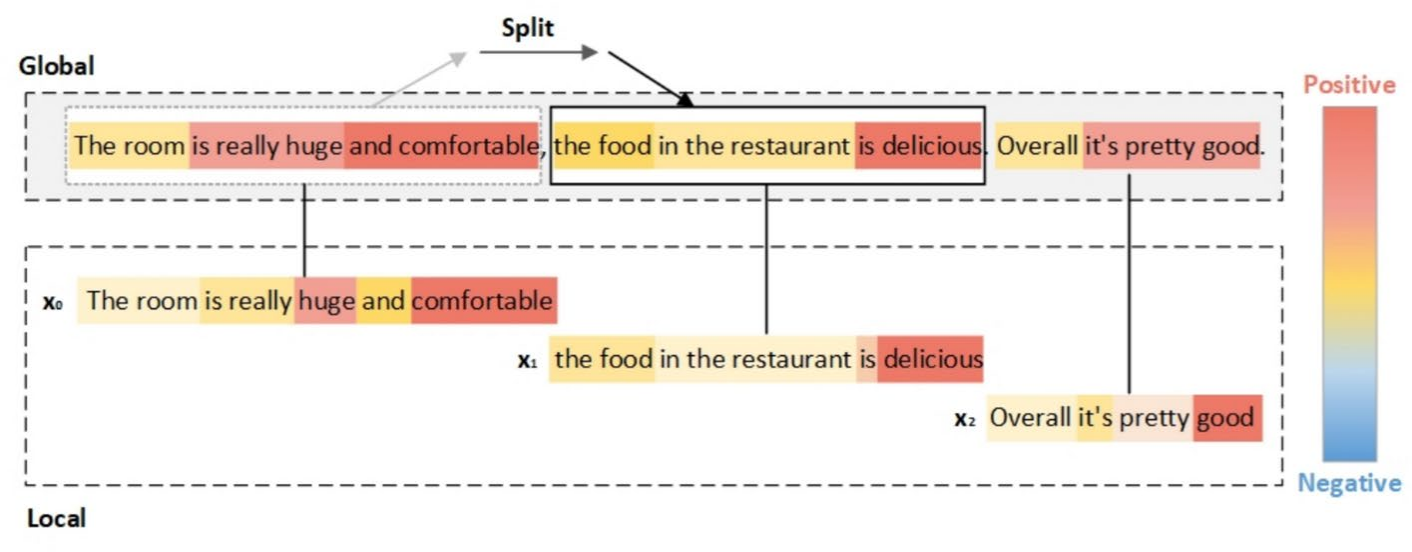
Comparison of global and local modeling. The example given is a positive sample “The room was exceptionally large and comfortable, the restaurant food was delicious, and overall it was okay” that from the Tansombo Hotel Review dataset. The contribution of each word is visualized using a blue-to-red heatmap, and the difference between global and local modeling is represented by different windows.

Unless otherwise specified, TextCNN is leveraged as the default feature extractor to evaluate the weights of sentence semantics, and the color of the word indicates the degree of its contribution to the final classification result. Where the color indicates the degree of positive samples of the word and the blue color indicates the degree of negative samples. Fig. 2 shows, that with global modeling (TextCNN), the semantic weights are evenly distributed across the sentence. In contrast, adaptive segmentation focuses locally within a sliding window, emphasizing keywords while filtering lexical noise. As we can see, the weight distribution of each word on sentence semantics is relatively uniform in global modeling. Whereas with adaptive segmentation, more attention can be paid to the respective local text, and the keywords can be made more prominent by filtering out some lexical noises that are not useful for semantic understanding. Therefore, by adjusting the segmentation window to focus locally, the adaptive text segmentation layer can discover and emphasize those semantic keywords that may be overlooked in the global modeling based on the whole sentence, and also filter out some of the noisy words. This provides a more powerful semantic representation for processing and understanding variable-length text.

Specifically, when the Pos-Embed obtains $x_i$ from the input layer, the dimension of the word vector is *Embed*, and the embedding matrix $X_i \in \mathbb {R}^{Seq \times Embed}$ of $x_i$ is obtained after encoding of the word vector. The feature extractor $F_{GE}$ extracts the features $f_{GE}$ from $X_i$ in the form of n-grams, and its formula is as follows:

1 $$\begin{aligned} f_{GE} = F_{GE}(X_i) = [c_0,c_1,c_2,...,c_{Seq-n}] = [c_j]_{j=0}^{Seq-n}, \end{aligned}$$

where $c_j$ denotes the convolution result of TextCNN for $X_i[j:j+n-1]$, *n* is the convolution kernel width, the convolution kernel size is $W \in \mathbb {R}^{n \times Embed}$, and the convolution bias is $b \in \mathbb {R}$. $c_i$ can be expressed as:

2 $$\begin{aligned} c_j = Func(W-X_i[j:j+n,:]+b)_{j=0}^{Seq-n}, \end{aligned}$$

where *Func* is a nonlinear activation function such as ReLU and GeLU. Then a maximum pooling operation is performed to obtain $f_{GE} = MaxPooling(f_{GE})$. the features extracted from $F_{GE}$ and $F_{LA}$ are referred to as respectively:

3 $$\begin{aligned} f_{GE} = TextCNN_{GE}(X), \end{aligned}$$

4 $$\begin{aligned} f_{LA} = TextCNN_{LA}(X), \end{aligned}$$

The features are then augmented and interacted with using the IE-Gate to obtain the final interaction semantic feature $f_{AFIE}$:

5 $$\begin{aligned} f_{AFIE} = IE-Gate(f_{GE}, f_{LA}). \end{aligned}$$

### GE-Net

The global feature extraction network GE-Net aims to learn the global semantic information of the input text to provide overall semantic features with a high degree of freedom and flexibility. GE-Net consists of an input layer Pos-Embed and a feature extractor $F_{GE}$. The input of GE-Net is the text $x_i$ with the length of *Seq*, and for the non-BERT model, firstly, the Pos-Embed is utilized to each word in the text is mapped to a k-dimensional continuous space to get the encoded-word embedding matrix $X_i \in \mathbb {R}^{Seq \times Embed}$. Then the feature extractor $F_{GE}$ (TextCNN, LSTM, and Transformer, *etc*.) is used on the embedding matrix to get the semantic features $f_{GE}$.

6 $$\begin{aligned} f_{GE} = F_{GE}(X), \end{aligned}$$

To capture text features at different scales, we introduce a multi-scale feature fusion mechanism:

7 $$\begin{aligned} f_{MS} = \sum _{i=1}^m(\alpha _i \times C_i), \end{aligned}$$

where $C_i$ is the convolution feature at the *i*-th scale, and $\alpha _i$ is the weight coefficient learned through an attention mechanism.

GE-Net provides a customizable framework for learning global semantic information using different feature extractors and representation models.

### LA-Net

Local feature extraction network LA-Net with adaptive text length aims to learn the local semantic information of the text. Through the adaptive text length for text cutting, the model focuses on the local features of the text. LA-Net consists of three parts: the input layer Pos-Embed, the adaptive text segmentation layer Split, feature extractor. Firstly, the text length is judged and segmented, and then the adaptive length is used for feature extraction using a classifier. Specifically first designed adaptive text segmentation layer Split to segment the text, defined as follows:

8 $$\begin{aligned} Split(X_i,L) = [x_0,x_1,x_2,...,x_k] = [x_k]_{k=0}^{Num-1} \end{aligned}$$

**Fig. 3:**
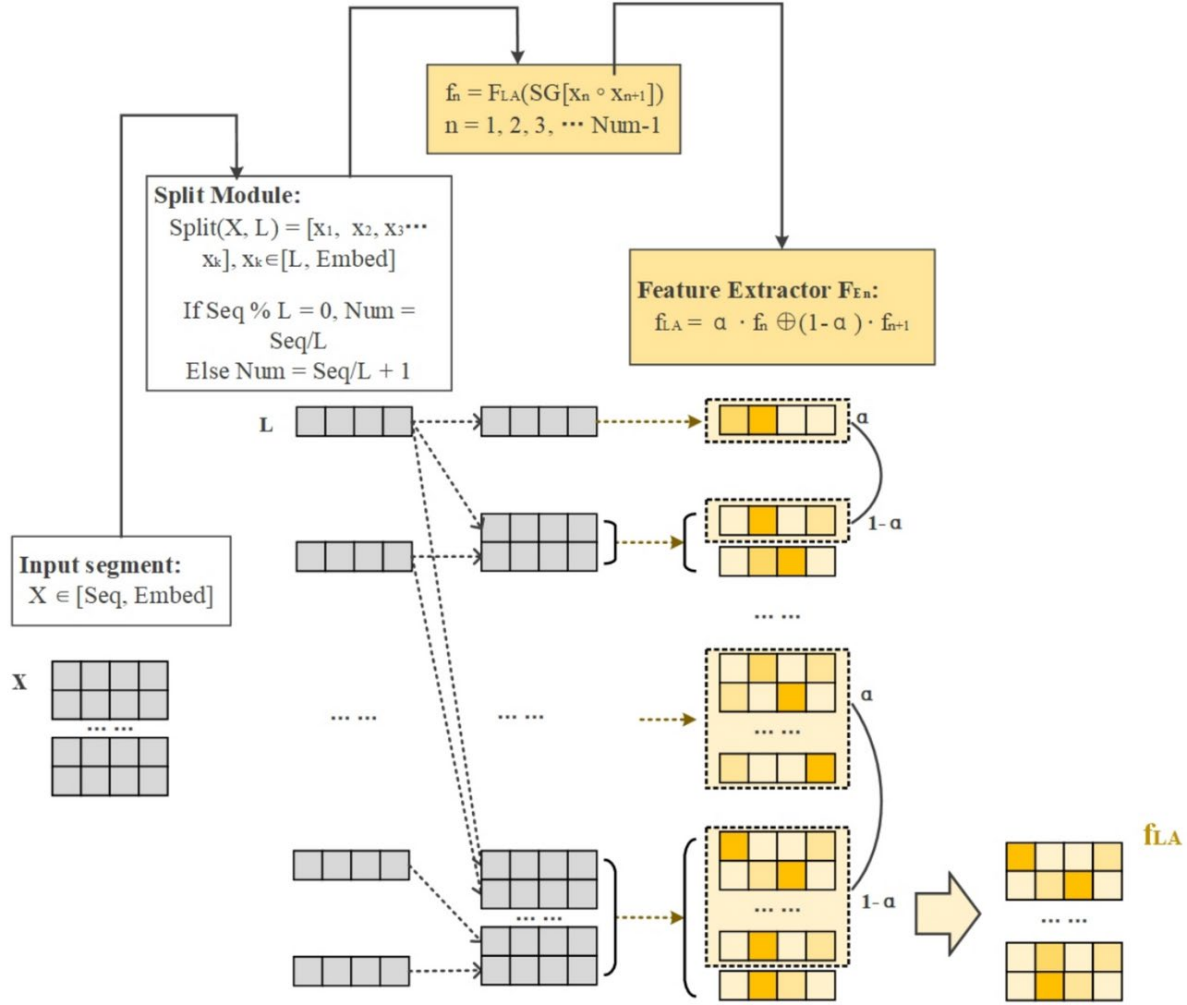
LA-Net local feature extraction network. We used different degrees of yellow color to represent the different weights of the individual words in the sentence.

LA-Net for the segmented text and then semantic information learning, the realization principle is shown in Fig. 3.

For the input text, the embedding matrix $X_i \in \mathbb {R}^{Seq \times Embed}$ is obtained after encoding, and $Set \in \mathbb {R}$ is set to set the length of the text for judging the length of the text, and $X_i$ is partitioned into *Num* matrices with a window of length *L*. The principle is as follows:

9 $$\begin{aligned} L = \left\{ \begin{aligned}&10, Seq \le Set,\\&20, Seq > Set, \end{aligned} \right. \end{aligned}$$

10 $$\begin{aligned} Num = \left\{ \begin{aligned}&Seq/L, Seq \% L = 0, \\&Seq/L+1, Seq \% L \ne 0. \end{aligned} \right. \end{aligned}$$

The partitioned matrix is denoted $X_i=[x_0,x_1,x_2,...,x_k]=[x_k]_{k=1}^{Num-1}$, where $[x_k]_{k=1}^{Num-1} \in \mathbb {R}^{(Seq\%L) \times Embed}$, the dimension size of $x_{k-1}$ is:

11 $$\begin{aligned} \left\{ \begin{aligned}&x_{k-1} \in \mathbb {R}^{L \times Embed}, Seq\%L=0, \\&x_{k-1} \in \mathbb {R}^{(Seq\%L) \times Embed}, Seq\%L \ne 0. \end{aligned} \right. \end{aligned}$$

The feature extractor $F_{LA}$ is used to compute the feature values of each part after segmentation, in the computation, the feature value $f_n$ of $x_n$ is obtained firstly, in the computation of the feature $f_{n+1}$ of $x_{n+1}$, $x_n$ is spliced with $x_{n+1}$ firstly and then computed, and the feature value of $x_{n+1}$ is fused with $f_n$ with the fusion ratio of $\alpha$ to get the local semantic feature $f_{LA}$ finally.

12 $$\begin{aligned} f_0 = F_{LA}(x_n),n=0, \end{aligned}$$

13 $$\begin{aligned} f_n = F_{LA}(SG[x_{n-1} \circ x_n]),n=0, \end{aligned}$$

14 $$\begin{aligned} f_n = \alpha \cdot f_{n-1} \oplus (1-\alpha ) \cdot f_n, (n \in \{0,1,2,...,Num-2\}). \end{aligned}$$

To better capture the semantic structure of the text, we introduce a dynamic segmentation method based on semantic similarity. It ensures that segments are contextually meaningful, facilitating the extraction of fine-grained local features. Defining a semantic similarity function $S(w_i,w_j)$, the set of segmentation points P can be represented as:

15 $$\begin{aligned} P = \{p_1,p_2,...,p_k\}, \end{aligned}$$

where $p_i = {\arg \min }_j \, S(w_j,w_{j+1})$, *k* is the number of segments dynamically determined based on the text length. Adaptive segmentation method allows LA-Net to generalize well to various text lengths and domains. This adaptability minimizes information loss for shorter texts while preventing feature oversaturation for longer texts. For instance, in tasks with short texts (*e.g.*, sentiment classification), smaller window sizes improve local feature granularity, leading to better performance. Conversely, for longer documents (*e.g.*, topic classification), larger window sizes and dynamic segmentation ensure that global coherence is preserved.

### IE-Gate

In this article, we propose a gating mechanism for the fusion of global and local features. Specifically, we use the confidence of global features to evaluate the filtering, and only select the global semantics with higher confidence as the fusion input. This design can serve to filter noise and avoid the interference of irrelevant and invalid information in local features on the final semantics. At the same time, we use the confidence of global features as the weight of fusion to dynamically regulate the proportion of different semantic representations and realize the adaptive processing of long and short texts. In this way, we play the role of the global features’ orientation and also retain the effective supplementation of local information.

IE-Gate is designed to selectively implement local feature enhancement by finding the part of features that need to be enhanced more accurately and rationally through confidence evaluation. IE-Gate contains a component gate and classifier $C_{AFIE}$, which first performs confidence evaluation to get the distribution of confidence intervals of the global features, and then uses the local features to enhance the global features within the target confidence intervals, thus helping the classification. GE-Net extracts to the initial global feature $f_{GE}$ of the text and LA-Net adaptive text length extracts to the local feature $f_{LA}$, in order to utilize the two flexibly, the gate component is designed to fuse $f_{GE}$ and $f_{LA}$ using the VALUE function to obtain the interactive semantic feature $f_{AFIE}$.

16 $$\begin{aligned} \tilde{f_{GE}} = \sigma (f_{GE}), \end{aligned}$$

17 $$\begin{aligned} \left. \begin{aligned} \tilde{f_{AFIE}}&= IE-Gate(f_{GE},f_{LA},\beta ),\\&= \tilde{f_{GE}} \odot (f_{LA} \circ \beta ) \oplus f_{GE}, \end{aligned} \right. \end{aligned}$$

where $\sigma (\cdot )$ is a Sigmoid function to evaluate the confidence of the global feature $f_{AFIE}$, and the $\beta$ parameter functions as a memory gate that selectively integrates local feature enhancements based on the evaluated confidence of global features. For instance, when $\beta$ is close to 0, all enhanced information is retained, whereas a higher $\beta$ filters out potential noise, relying more on the original global features. This selective enhancement, controlled by the $\beta$-adjusted value function, ensures that noise-prone features are filtered out, improving the robustness and efficiency of the interaction. Attention is used in the classifier $C_{AFIE}$ to combine the interaction semantic features $f_{AFIE}$ and global features $f_{GE}$ for classification to the final label $Y_{Pred}$.

18 $$\begin{aligned} \left. \begin{aligned} Y_{Pred}&= Attention(f_{GE},f_{AFIE}),\\&= Softmax(f_{GE},f_{AFIE}^T) \cdot f_{AFIE}, \end{aligned} \right. \end{aligned}$$

If $\beta =0$, discard all enhanced information, then self-attention is computed at this point. After passing through the *softmax* layer, the feature vector is mapped to the label classification to compute the loss. An optimizer is used to minimize the cross-entropy loss *L*.

19 $$\begin{aligned} L = CrossEntropy(Y_{Truth}, Y_{Pred}), \end{aligned}$$

To enhance the model’s discriminative ability, we introduce the concept of contrastive learning and design a new loss function:

20 $$\begin{aligned} L = L_{CE}(f(x),y)+\lambda L_{contrastive}, \end{aligned}$$

where $L_{CE}$ is the cross-entropy loss and $L_contrastive$ is the contrastive loss. To achieve more fine-grained feature interaction, we introduce an interaction enhancement module based on multi-head attention:

21 $$\begin{aligned} H = MHA(F_G,F_L,F_L) \end{aligned}$$

22 $$\begin{aligned} G=\sigma (W_g[F_G; H] + b_g) \end{aligned}$$

23 $$\begin{aligned} F_E = G \odot F_G+(1-G) \odot H \end{aligned}$$

where *MHA* represents the multi-head attention function, $F_G$ and $F_L$ are global and local features respectively, and $W_g$ and $b_g$ are learnable parameters.

Unlike conventional attention mechanisms that assign weights based on direct feature similarities, the IE-Gate introduces a confidence evaluation attention mechanism, which dynamically evaluates the reliability of global features ($F_G$) and selectively enhances them using local features ($F_L$). The incorporation of the confidence parameter $\beta$ enables the model to adaptively balance augmentation and noise suppression.

To facilitate understanding of the above process, we summarize the entire process of AFIE-Net as Algorithm 1, which includes the correspondence between each step and the previous description.

**Algorithm 1:**
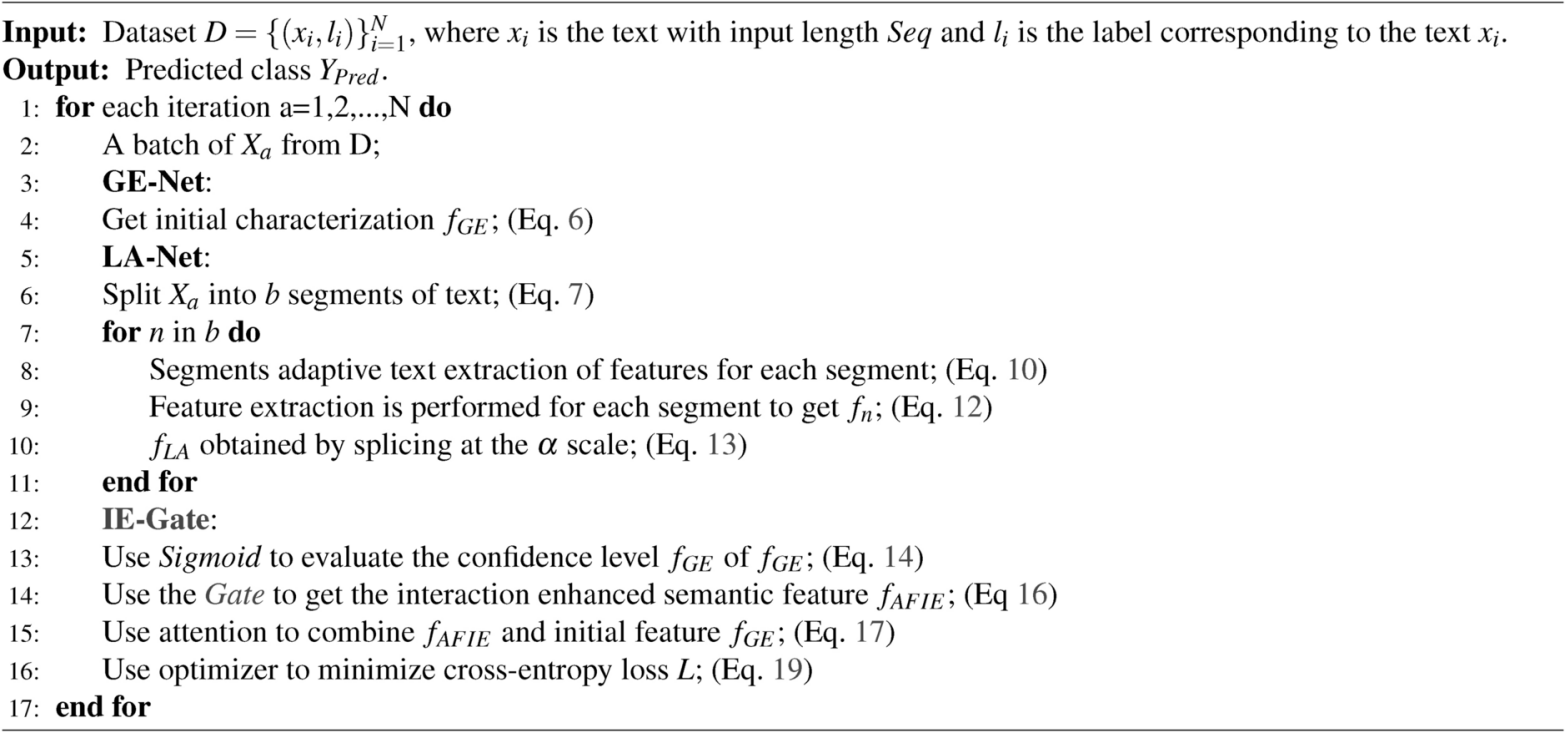
Adaptive Feature Enhancement Network (AFIENet)

## Experimental procedure and analysis

### Dataset

Our experimental dataset consists of two public datasets and one self-constructed dataset, the details of the dataset are shown below:

**THUCNews**: a dataset used for news classification, involving finance and economics, real estate, stocks, education, science and technology, society, current affairs, sports, games, and entertainment, a total of 10 categories, each category of 20,000, a total of 200,000, with an average data length of 22.34 words, belonging to the category of a balanced distribution of fine-grained short text dataset.

**ChnSentiCorp**: a hotel review dataset, divided into 2 categories of positive and negative reviews totaling 7,765 pieces of data, of which 5,322 positive reviews and 2,443 negative reviews, with an average data length of 128.52 words, belonging to the coarse-grained long text dataset with unbalanced distribution of categories.

**ChemicalPro**: To better reflect the effectiveness of the model on specialized domain text data, the Chinese domain text we used is the self-constructed chemical domain text dataset. The chemical domain text data is characterized by high specialization and sparse association between words and meanings, which can better reflect the characteristics of domain class text data. The data is collected from chemical product information from several major domestic chemical trading platforms such as China Chemical Manufacturing Website, Chemical Products Website, and Gaide Chemical Website. The dataset has a total of 128,053 samples, which belong to 17 categories, such as organic chemicals, inorganic chemicals, chemical intermediates, and biochemical. The average words length of the dataset is 533.77, and the number of samples of the categories is unevenly distributed.

### Implementation details

To validate the effectiveness of our proposed method, experimental analyses are carried out using the following model as a feature extractor, respectively.

**TextCNN**^7^: a convolutional neural network model proposed by Kim et al. in 2014. It extracts the local features of text through convolutional kernels of different sizes and fuses them to get the text representation. TextCNN is a typical CNN-based text feature extractor.

**DPCNN**^8^: a convolutional neural network-based text classification model proposed by Johnson in 2017, which constructs text representations through region-level supervision.

**LSTM**^11^: a text classification model named long and short-term memory network proposed by Liu et al. in 2016, in order that long-range sequential information can be preserved in text modeling.

**Transformer**^13^: an encoding model of the full attention mechanism proposed by Vaswani et al. in 2017, with the advantages of parallel computing and long-range dependency modeling.

**MacBERT**^32^: a Chinese pre-trained language model proposed by Cui et al. in 2020. It pre-trains and encodes semantic knowledge on large-scale Chinese corpus. MacBERT is one of the most effective Chinese text feature extraction models.

The specific configuration of the above feature extractors is as follows:

1. DPCNN: 250 convolutional filters are used with filter sizes [3, 4, and 5].
2. TextCNN: we use a filter size of [2, 3, and 4] with a number of hides set to 250.
3. RNN: a 3-layer LSTM is used as the feature extractor with the number of hidden ones set to 256 for each.
4. Transformer: 1 layer of Transformer’s encoder is used as the feature extractor.
5. MacBERT: the sum file with its own pre-trained parameters is used.

In the LA-Net network, the length of the text is set to 50, and the feature fusion ratio $\alpha$ after text segmentation is set to 0.5. In IE-Gate, the *Gate* function sets the memory parameter $\beta$ for the fusion of $f_{GE}$ and $f_{LA}$ to 0.5.

### Evaluation metrics

In the experiment, Precision (P), Accuracy (A), and F1-score are used as evaluation indexes to evaluate the classification effect of the model. The Precision is the proportion of correctly predicted samples out of all samples predicted to be positive cases, a higher Precision indicates that the model is making fewer false positive predictions, which is particularly critical in scenarios where the cost of false positives is high. The value of Precision reflects the model’s ability to accurately identify relevant instances. The formula is calculated as:

24 $$\begin{aligned} P = \frac{TP}{TP+FP} \end{aligned}$$

The Accuracy refers to the ratio of the number of correct samples predicted by the model to the total number of samples, which provides an overall measure of the model’s correctness across all predictions. The formula is calculated as:

25 $$\begin{aligned} A = \frac{TP+FN}{TP+TN+FP+FN} \end{aligned}$$

The Recall refers to the proportion of samples that are correctly predicted out of all samples that are true as positive examples, a higher Recall demonstrates the model’s capacity to identify more relevant instances from the dataset, reducing false negatives, which is important in applications where missing relevant instances is costly.. The formula is calculated as:

26 $$\begin{aligned} R = \frac{TP}{TP+FN} \end{aligned}$$

The F1-score value is used to combine precision and recall for a comprehensive evaluation of model effectiveness, which balances Precision and Recall, providing a comprehensive view of the model’s performance, particularly when there is an imbalance between false positives and false negatives. The formula is calculated as:

27 $$\begin{aligned} F1 = 2 \times \frac{P \times R}{P + R} \end{aligned}$$

In the formula for the above evaluation metrics, TP, FP, TN, and FN denote ture positive, false positive, true negative, and false negative in predictions.

## Analysis of experimental results

### Model validity assessment

Considering the generality, validity, and accuracy of the proposed method for different datasets and different classification models, the accuracy rate and F1-score value are used as evaluation metrics in the experiments. The experimental results are shown in Table 1. The experiments compare the effectiveness of classification using TextCNN, DPCNN, LSTM, and Transformer models along with the effectiveness of model classification with the addition of the AFIENet network. The experiments use static word vectors obtained from pre-training with Glove^37^ as word embeddings.

**Table 1:** Performance comparison of AFIENet combined with different backbone network on THUCNews, ChnSentiCorp, and ChemicalPro datasets (%).

Method	THUCNews		ChnSentiCorp		ChemicalPro		Average	
	Accuracy	F1	Accuracy	F1	Accuracy	F1	Accuracy	F1
TextCNN	91.02	91.03	85.51	85.30	86.45	86.44	87.66	87.59
AFIE-TextCNN	**97.55**	**97.55**	**88.64**	**88.65**	**88.24**	**88.20**	**91.48**	**91.47**
DPCNN	91.15	91.12	84.93	84.83	85.72	85.63	87.27	87.19
AFIE-DPCNN	**93.62**	**93.59**	**86.05**	**86.09**	**87.86**	**87.82**	**89.18**	**89.17**
LSTM	90.99	91.00	83.52	83.38	81.88	82.01	85.46	85.46
AFIE-LSTM	**91.60**	**91.56**	**85.45**	**85.12**	**84.52**	**84.42**	**87.19**	**87.03**
Transformer	89.73	89.74	82.42	81.90	78.59	78.49	83.58	83.38
AFIE-Transformer	**90.01**	**90.01**	**83.13**	**82.87**	**81.37**	**81.29**	**84.84**	**84.72**

AFIE-* indicates that both GE-Net and LA-Net in AFIE-Net use * as the feature extractor.

Models * and AFIE - * form a comparison group. The bold is used to mark the group with better experimental performance in this comparison.

1. The experimental results show that the average accuracy of TextCNN, DPCNN, LSTM, and Transformer using the AFIENet network on the three datasets is improved by 3.82, 1.63, 1.73, and 1.26 percentage points, respectively, compared with the original model, which validates the effectiveness of the proposed method. The analysis reveals that GE-Net can extract the global semantic features of the text, and LA-Net can learn segmented features according to the length of the text. Thus models using AFIENet can focus on more local feature information, thus improving model accuracy. For models with strong local feature extraction, AFIENet enhances their global modeling capabilities. For models with strong global feature extraction capability, the segmented feature interaction mechanism in AFIENet can enhance its local feature learning capability. Therefore, AFIENet can significantly improve the effectiveness of different feature extractors on multiple datasets. In particular, the performance improvement is more significant when TextCNN, which is strong in joint local feature learning, is used as a feature extractor.
2. On the THUCNews dataset, the three models improved their accuracy by 2.47 percentage points on average. The average improvement on the ChnSentiCorp dataset was 1.72 percentage points. The average improvement on the ChemicalPro dataset is 2.35 percentage points. THUCNews and ChemicalPro are fine-grained datasets that improve even more with the AFIENet network. This is due to the large differences between fine-grained text categories, and LA-Net segmented feature extraction can find effective features. ChnSentiCorp is a coarse-grained dataset with a small number of texts and little variation. Although LA-Net can also focus on local information, the enhancement is not obvious.
3. Although AFIE-TextCNN uses only the combined structure of TextCNN and AFIENet, it achieves good results on the THUCNews dataset. Analyzing the reason, TextCNN extracts features using convolutional kernels of different sizes and focuses more on local information compared to other models. THUCNews is a short text dataset with dense information, and the local modeling capability of TextCNN is fully exploited. Both GE-Net and LA-Net in AFIENet use TextCNN to enhance global and local feature learning. So the fusion of TextCNN with AFIENet can improve the performance on short text datasets such as THUCNews. In addition, AFIE-TextCNN achieves good results on the ChemicalPro dataset.

**Fig. 4:**
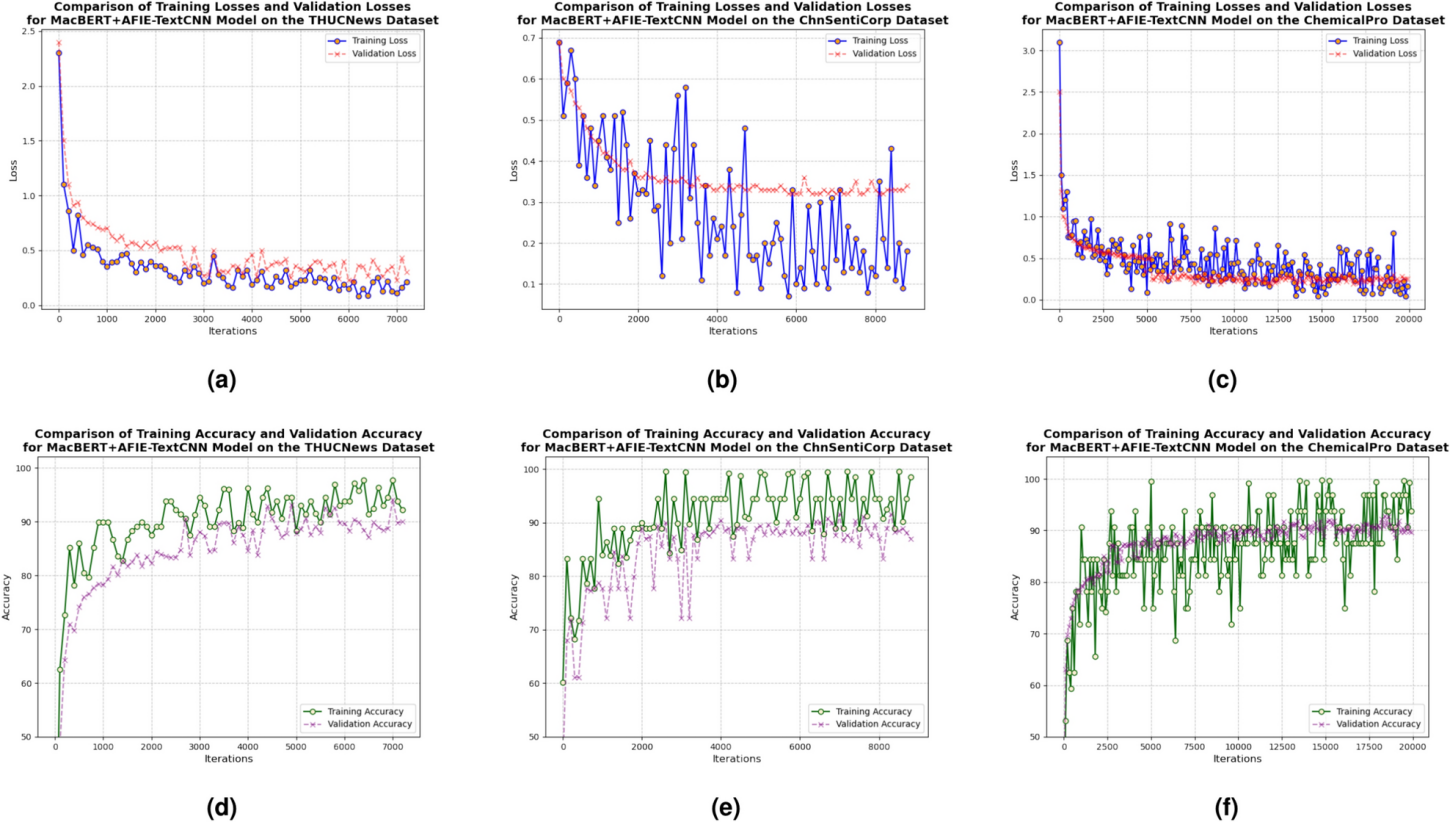
Visualization of training process on THUCNews, ChnSentiCorp, and ChemicalPro datasets, respectively.

We compared the performance of the MacBERT-only model, the model with AFIE-* after pre-training on MacBERT, on multiple datasets. The variation trends of training and validation loss, classification accuracy, and F1-score during the training process are shown in the Fig. 4. The results of the experiments are shown in Table 2. Five independent experiments were repeated for each AFIE-* model and the mean and standard deviation of the indicators obtained.

Table 2 shows that the MacBERT model has an average accuracy of 91.24% on the three datasets THUCNews, ChnSentiCorp, and ChemicalPro. After fusing the model with the AFIE module on top of the MacBERT pre-training, it improves the average accuracy to 92.68%, 90.49%, and 88.20%, respectively. In particular, when TextCNN is used as the feature extractor, the Mac-BERT + AFIE-TextCNN shows varying degrees of accuracy improvement over MacBERT for all three datasets. Accuracy improved by an average of 1.44 percentage points. This indicates that the AFIENet we proposed can effectively enhance the text modeling capability of the model and can incorporate the advantages of pre-training. The model did not improve much on the ChnSentiCorp dataset. The reason for this problem is that the ChnSentiCorp training set has a small sample size and the model is not adequately trained.

**Table 2:** Performance comparison of AFIENet pre-trained on MacBERT (%).

Model name	THUCNews		ChnSentiCorp		ChemicalPro	
	Accuracy	F1	Accuracy	F1	Accuracy	F1
MacBERT	94.56	94.56	**90.60**	**90.60**	88.57	88.49
AFIE-TextCNN	97.55	97.55	88.64	88.65	88.24	88.20
MacBERT + AFIE-TextCNN	**97.91** (±0.3)	**97.95 **(±0.3)	**89.97 **(±0.2)	**89.95 **(±0.2)	**88.68 **(±0.1)	**88.68** (±0.1)
AFIE-DPCNN	93.62	93.59	86.05	86.09	87.66	87.62
MacBERT + AFIE-DPCNN	**94.32 **(±0.2)	**94.33** (±0.2)	**87.41 **(±0.1)	**87.45** (±0.1)	**88.56** (±0.3)	**88.56 **(±0.3)
AFIE-LSTM	91.60	91.56	85.45	85.12	84.52	84.42
MacBERT + AFIE-LSTM	**92.12** (±0.2)	**92.11** (±0.2)	**85.97** (±0.1)	**85.99** (±0.1)	**85.37 **(±0.2)	**85.36 **(±0.2)

AFIE-* indicates that both GE-Net and LA-Net in AFIE-Net use * as the feature extractor.

Models AFIE - * and MacBERT + AFIE - * serve as a comparison group. The result of the group with better experimental performance is also highlighted in bold.

### Model generalization assessment

We tested the model inference speed under the same hardware conditions, the GPU model is “NVIDIA Quadro RTX4000” and the CPU model is “Intel(R) Xeon(R) Silver 4210R”. TextCNN, DPCNN, LSTM, Transformer, the model after using the AFIENet network, and the MacBERT model were selected for the experiments to compare their effects on the THUCNews dataset. The experiments also calculated the number of parameters and the speed of inference for each method. AFIE-* is a model for the proposed AFIENet network in which both GE-Net and LA-Net use * as a feature extractor. The results of the models in terms of the number of parameters and the speed of reasoning compared to the model after using the AFIENet network are given in Table 3, in which M stands for million and ms/it stands for the time spent reasoning about each piece of data.

As we can see in Table 3, the number of parameters in AFIENet increased by up to 4% compared to the original model, but the accuracy improved by up to 6.52 percentage points. The reason for this phenomenon is that AFIENet does not change the structure of the original model and only enhances the representation through adaptive length local feature extraction and global feature interaction. Thus performance can be effectively improved with less impact on the original model. In terms of inference time, the AFIENet network reduces it by about 100 percent on average. However, since the inference time of the original model is then shorter, the absolute inference speed of the AFIE model is still faster and still meets the needs of practical applications. AFIE-TextCNN has only 2.1% of the number of parameters of MacBERT, but the inference speed is 4.64 times faster than MacBERT, and the accuracy is also improved by 2.99 percentage points. The main reason for this phenomenon is the faster raw inference time of the network, which maintains its speed advantage after using AFIENet.

**Table 3:** Comparison of number of parameters and inference speed of different models.

Model name	Number of parameters	THUCNews	
		Inference speed (ms/it)	F1 (%)
TextCNN	2.13M (1$\times$)	5.52	91.03
AFIE-TextCNN	**2.14M **(1.01$\times$)	**9.10**	**97.55**
DPCNN	2.21M (1$\times$)	5.17	91.15
AFIE-DPCNN	**2.25M** (1.02$\times$)	**8.65**	**93.62**
LSTM	5.04M (1$\times$)	2.27	91.00
AFIE-LSTM	**5.24M **(1.04$\times$)	**6.53**	**91.56**
Transformer	3.48M (1$\times$)	3.68	89.74
AFIE-Transformer	**3.49M **(1.01$\times$)	**7.32**	**90.01**
MacBERT	102.9M	42.25	94.56

Models * and AFIE - * form a comparison group. The bold is used to mark the group with better experimental performance in this comparison.

### Comparison with state-of-the-art methods

In order to show the progressiveness of the method proposed in this article, we compared the results of state-of-the-art (SOTA) text classification methods in the past few years. Specifically, for backbone networks such as TextCNN, DPCNN, LSTM, and Transformer, the results when using them alone are presented. In addition, performance of SOTA methods including RBTL3, ELECTRA, BERT-CNN, and ERNIE are reported for comparison. The comparison results are shown in Table 4, which shows that our proposed AFIE-Net has the best performance when using Text CNN as the backbone network, and is also very competitive when using other backbone networks.

**Table 4:** Performance comparison results with state-of-the-art methods.

Model name	THUCNews		ChnSentiCorp		ChemicalPro	
	Accuracy	F1	Accuracy	F1	Accuracy	F1
TextCNN	91.02	91.03	85.51	85.30	86.45	86.44
DPCNN	91.15	91.12	84.93	84.83	85.72	85.63
LSTM	90.99	91.00	83.52	83.38	81.88	82.01
Transformer	89.73	89.74	82.42	81.90	78.59	78.49
RBTL3	93.30	93.29	86.50	86.52	85.32	85.33
ELECTRA	93.68	93.68	88.53	88.51	84.79	84.78
BERT-CNN	91.28	91.26	89.17	89.15	85.91	85.88
ERNIE	94.07	94.08	89.37	89.37	86.09	86.11
AFIE-TextCNN	**97.55**	**97.55**	**88.64**	**88.65**	**88.24**	**88.20**
AFIE-DPCNN	93.62	93.59	86.05	86.09	87.86	87.82
AFIE-LSTM	91.60	91.56	85.45	85.12	84.52	84.42
AFIE-Transformer	90.01	90.01	83.13	82.87	81.37	81.29

The bold marks the group with the optimal experimental performance among all models, aiming to highlight the best experimental result.

### Ablation study

To illustrate the role of each part of the feature enhancement network AFIENet more effectively. We conducted ablation experiments on the THUCNews dataset, using F1-Score as the evaluation metric. The experimental results are shown in Table 5. Where the feature extractors $f_{GE}$, $f_{LA}$ in GE-Net and LA-Net use TextCNN, DPCNN, 3-layer LSTM, and Transformer, respectively. The experiments compare several scenarios: using GE-Net only, using GE-Net and LA-Net but not IE-Gate, using GE-Net, LA-Net and IE-Gate, using GE-Net, LA-Net and IE-Gate.

As can be seen from the experimental results in Table 5, relative to the control groups A, B, C, and D, the ablation experiments of the A1st, B1st, C1st, and D1st groups made the F1-Score decrease by 6.52, 2.5, 0.56, and 0.27 percentage points, respectively. This is mainly due to ignoring the local features of LA-Net, which leads to the lack of keyword information, resulting in poorer model experiment results. Meanwhile, the ablation experiments of groups A2, B2, C2, and D2 made the F1-Score decrease by 6.74, 2.65, 0.87, and 0.21 percentage points, respectively, which showed a large decrease. It was analyzed and learned that this is because IE-Gate selects the enhanced local features based on the global features of GE-Net, and if this mechanism is not used, the two are simple feature splicing, which introduces unnecessary perturbations.

To further validate the ability of each model for adaptive feature extraction in the local feature extraction network LA-Net, we conducted experiments on the THUCNews dataset using different models as feature extractors in LA-Net. The results of their experiments are shown in Table 5, where accuracy and F1-score value were used as evaluation metrics. Where LA-* denotes the use of * as a feature extractor for LA-Net, TextCNN, DPCNN, LSTM with 2 and 3 layers, and Transformer were selected.

**Table 5:** Ablation study of the proposed GE-Net, LA-Net, and IE Gate (%).

Model name	Group	GE	FLA	Gate	F1
TextCNN	Group A	$\checkmark$	$\checkmark$	$\checkmark$	**97.55**
	A1	$\checkmark$	–	–	91.03
	A2	$\checkmark$	$\checkmark$	-	90.81
DPCNN	Group B	$\checkmark$	$\checkmark$	$\checkmark$	**93.62**
	B1	$\checkmark$	–	–	91.12
	B2	$\checkmark$	$\checkmark$	–	90.97
LSTM (3)	Group C	$\checkmark$	$\checkmark$	$\checkmark$	**91.56**
	C1	$\checkmark$	–	–	91.00
	C2	$\checkmark$	$\checkmark$	–	90.69
Transformer	Group D	$\checkmark$	$\checkmark$	$\checkmark$	**90.01**
	D1	$\checkmark$	–	–	89.74
	D2	$\checkmark$	$\checkmark$	–	89.80

In the ablation experiment with "Model name" as a group, the result with the optimal performance in this group is emphasized in bold.

As we can see from Table 6, the best accuracy of 91.54% was achieved using TextCNN as the feature extractor for LA-Net. In contrast, the results of the remaining models are slightly lower. The analysis reveals that this is mainly due to the fact that THUCNews belongs to a short text dataset with a concentrated feature distribution. In this scenario, local feature extraction with TextCNN after adaptive segmentation can achieve a better representation.

**Table 6:** Performance comparison of LA-Net using different feature-enhanced networks (%).

Model name	THUCNews	
	Accuracy	F1
LA_TextCNN	**91.54**	**91.54**
DPCNN	91.43	91.42
LA_RNN (2)	90.41	90.38
LA_RNN (3)	90.13	90.12
LA_Transformer	90.19	90.16

The experimental result of the group with the optimal performance is marked in bold.

## Conclusion

In this article, we propose an Adaptive Feature Interaction Enhancement Network (AFIENet) for text classification. The model utilizes an internal gating mechanism to incorporate both global and local key information for feature enhancement. Specifically, AFIENet contains two subnetworks for global and local feature learning. For local feature extraction, an adaptive text length-based network is proposed to focus on local contexts. Then, through the designed interaction enhancement gate, local features are selectively fused with global features based on the global feature confidence distribution generated by the gating mechanism. This achieves effective enhancement of global features.

Currently, research on local feature fusion through interaction enhancement gates is still rare. We validated the effectiveness of this fusion mechanism through comparative experiments. It should be noted that due to length limitations, only several typical models were selected for experimental verification. In future work, richer types of feature extraction networks could be explored, or AFIENet could be extended to other directions such as graph neural networks, *e.g.* graph convolutional networks and generative adversarial networks, to further enrich the application scenarios of this fusion mechanism.

## Data Availability

The datasets used and analyzed in the current study are available from the corresponding author upon reasonable request.
